# The (in)accuracies of floating leaves: How people with varying experiences of dementia differently position the same visual metaphor

**DOI:** 10.1177/14713012211072507

**Published:** 2022-02-11

**Authors:** Emma Putland

**Affiliations:** 6123 Lancaster University, UK

**Keywords:** dementia, metaphor, visual, brain, thematic discourse analysis, focus groups, interviews

## Abstract

Metaphors help shape the social world. Yet, with research and language guidelines focusing primarily on the stigmatising potential of verbal representations, much greater attention is needed regarding visual metaphors’ role in perpetuating and challenging particular views of dementia. Through semi-structured interviews and focus groups, this paper explores how people with dementia and their carers and/or loved ones evaluate one prevalent visual metaphor for dementia that maps autumnal trees losing leaves onto the brain/head. Analysis considers three main responses to the metaphor, that: (1) it does not depict dementia; (2) it meaningfully explains a biomedical account of progressive brain deterioration; and (3) it reinforces inaccurate and/or ‘hopeless’ discourses of what having dementia involves, with individuals suggesting creative alterations to better fit their counter discourses. These findings foreground the importance of attending to subjectivity, nuance and multi-layered discourses within visual metaphors, which can indirectly convey stigmatising representations.

## Introduction

Metaphors are important conveyors of social ideologies and can be expressed through different modes and with different degrees of explicitness. For instance, the prevalent and stigmatising metaphor that people with dementia are zombies can be conveyed linguistically, through both explicit references to people with dementia as ‘the living dead’ and subtler discussions of a loss of self (Behuniak, 2011). The metaphor can also be expressed through other modes, including visually representing people with dementia as vacant, incomplete and as ‘a “non-person” existing principally as corporeal matter’ (Harvey & Brookes, 2019, p. 997). Positioning people with dementia as sub-human has serious consequences, such as failing to respect people’s personhood (Kitwood, 1997) and violating individuals’ human rights (Cahill, 2018). Without a forthcoming cure, it is vital to address dementia socially, including through improving understandings and reducing stigma (Kenigsberg et al., 2016), so that no one need say that the ‘ugliest part of having dementia is probably the reaction of others’ (Swaffer, 2016, p. 66). Metaphors have a significant role to play in achieving this social shift.

A social constructionist stance acknowledges that metaphors help to shape cultural responses to dementia through either naturalising or challenging specific worldviews, making metaphors an important site for social change (Johnstone, 2013). Equally, it is widely acknowledged that appreciating and showcasing the expressions of people with dementia (e.g. through metaphor, poetry and art) can promote self-expression and improve others’ understandings (Gregory, 2014; Killick, 2017; Swinnen, 2016). Critiquing, contributing and developing metaphors must therefore happen in collaboration with people affected by dementia ― that is, people with dementia, as well as their carers, close family and friends (Bould, 2018). This will generate more empowering understandings and interactions that reflect the richer, more nuanced insight of direct experience.

It is consistently shown that the meanings of metaphors and images are highly subjective and contextual (ño, 2020; Pink, 2011; Winter et al., 2020). Existing research that incorporates the perspectives of people affected by dementia currently focuses upon researchers’ analyses of people’s verbal or written metaphorical expressions (Brown Wilson et al., 2021; Castaño, 2020; Golden et al., 2012; Johannessen et al., 2015; Peel & Harding, 2014; Thorsen & Johannessen, 2021; Zimmermann, 2017). Yet, metaphors are also manifest in a range of non-verbal communicative modes, including gesture, images, sound and film (Forceville & Urios-Aparisi, 2009; Semino & Demjén, 2016). Only recently has the role of visual metaphors in communicating and shaping understandings of dementia begun to be seriously considered (see Brookes et al., 2018; Caldwell et al., 2021; Harvey & Brookes, 2019; Schweda, 2019), and the author is currently not aware of any studies that directly consult people affected by dementia about this topic. Likewise, many influential guidelines on the language used when discussing dementia consult people mostly about verbal, rather than visual representations (Alzheimer’s Society, 2018; Bould, 2018; DEEP, 2014; YoungDementia UK, 2020). In a society that is increasingly saturated by, and literate in, visual communication (Ledin & Machin, 2018b), how people affected by dementia respond to visual metaphors requires much greater attention.

Acknowledging the range of theories on metaphor (see Semino & Demjén, 2016), this paper draws primarily on Cognitive Metaphor Theory (Lakoff & Johnson, 1980; Semino, 2008) and discourse approaches to metaphor (Grebe et al., 2014). According to Cognitive Metaphor Theory, metaphor provides us with the tools to make complex, abstract, unfamiliar, subjective and/or poorly defined phenomena more intelligible and communicable. This is achieved by mapping features of a ‘source domain’, which is often more concrete, familiar, simple, physical and/or a well-defined experience, onto a more complex or abstract ‘target domain’ (here, dementia) (Semino, 2008). A discourse analysis perspective further recognises metaphors’ ideological implications by positioning them as a framing discursive device. By discourse, I refer to a particular version of the world (e.g. that dementia is a biomedical disease versus a bio-psycho-social condition: Sabat, 2014), that is manifest in a set of resources (here images, metaphors and speech) (Burr, 2015) and that not only reflects but *shapes* social realities (Foucault, 1972). Metaphors frame social phenomena in line with their associated discourse(s) by foregrounding certain aspects of a scenario while downplaying or ignoring others, thereby promoting ‘a particular problem definition, causal interpretation, moral evaluation and/or treatment recommendation for the item described’ (Entman, 1993, p. 52).

Notably, in a Western context, fear-inducing discourses surrounding dementia are such that Zeilig (2014, p. 262) suggests that dementia has itself become a metaphorical device, whereby ‘Dementia = a complex, unknowable world of doom, ageing, and a fate worse than death’. Research indicates that popular metaphors often disempower, depersonalise and even dehumanise people with dementia, including by positioning people as zombies, failing machines, disappearing, empty shells, infants and a heavy burden (Alzheimer Europe, 2013; Bailey et al., 2021; Van Gorp & Vercruysse, 2012). These metaphors all contribute to an overall discourse of dementia as the loss of cognitive abilities and subsequently of someone’s self, which is grounded in a hypercognitive view of selfhood, perhaps more accurately termed ‘brainhood’: ‘the property or quality of *being*, rather than simply *having*, a brain’ (Vidal, 2009, p. 6, author’s emphasis). Countering this discourse is clear evidence that sense of self continues with dementia (Bryden, 2020) and the existence of more holistic models of selfhood and personhood (Hughes, 2014; Kitwood, 1997; Kontos, 2006; Sabat, 2018). Although guidance for best practice in representing dementia recognises the potential of metaphors to cause harm, the focus currently remains on linguistic, rather than visual, manifestations (Alzheimer’s Society, 2018; Bould, 2018; DEEP, 2014; YoungDementia UK, 2020).

**Figure 1.**
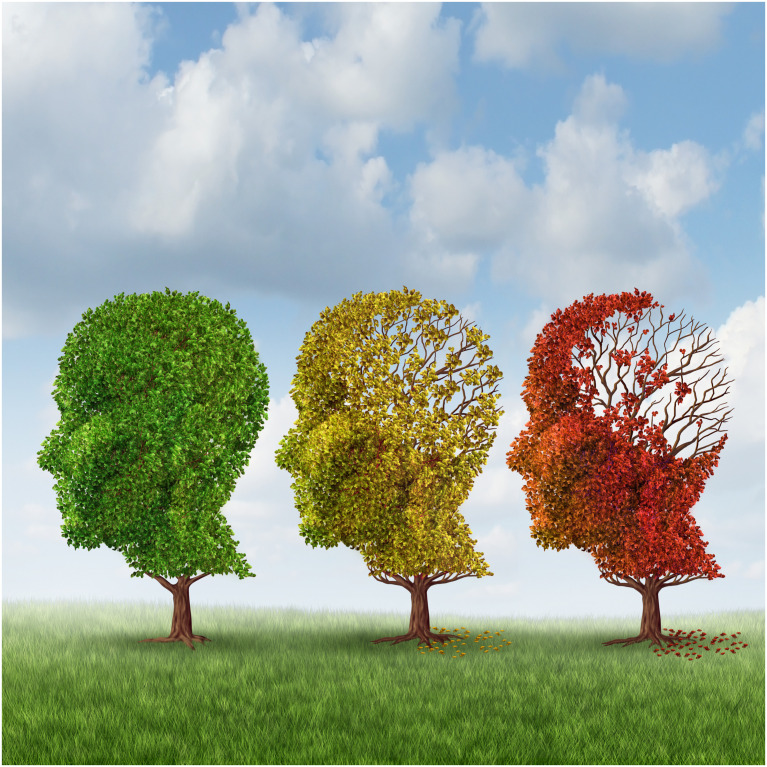
A visual metaphor shown to participants in focus groups and interviews. Reproduced according to Shutterstock’s standard royalty-free license.

In contrast, trees, as a ‘symbol of life and image of seasonal change’ (Zimmermann, 2017, p. 80), ostensibly offer a less stigmatising route to representing people with dementia. Tree metaphors are an important feature of people’s accounts of dementia (Zeilig, 2014a, 2014b; Zimmermann, 2017), to the point that Thelker’s (2018) blog for Dementia Alliance International is titled ‘Walking through the Neuron Forest ...called Dementia’. Thelker’s ‘Neuron Forest’ metaphor reflects the common trend to map trees/plants onto the brain. In an analysis of picture books, Caldwell et al. (2021) establish how a visual plant metaphor simplifies a biomedical explanation of dementia by using weeds growing in a garden to represent increasing tangles in the brain. Just as the weed illustration is evaluated as appropriate for young children, the visual metaphor that this paper examines (see Figure 1) is found across not only media articles (most recently, *The Conversation*: Weaver, 2021) but a range of materials advocating for or directed at people with dementia. This includes a book advancing a relational approach to personhood and rights (Hughes, 2014), a local activities information leaflet (Warwickshire County Council Localities team, 2017), and a university article about dementia awareness (Loughborough University, 2016). Despite its prominence and large, diverse audience, little is known about the reception of less openly stigmatising visual metaphors such as this.

This paper emerged from a larger project exploring how people with dementia, carers and family/friends situate themselves in relation to popular dementia discourses, which will be discussed further in the following section. The image focused on here garnered particular attention from participants and denotes three head-shaped trees in a line. Moving from left to right, the head-shaped trees change colour from green, to yellow, to red, with decreasing foliage where the brain is located. As will be discussed, this image engages with multiple metaphors, notably THE BRAIN IS A TREE and DEMENTIA IS LOSS/DECAY. Focusing on a single visual metaphor for this paper enables an in-depth examination of the following research questions:

1. How do different participants interpret the same visual metaphor?
2. How do participants situate the visual metaphor in relation to their own experiences and conceptualisations of what dementia means? How accurate and meaningful do they find this metaphor to be?
3. How do participants’ responses reproduce or challenge dominant discourses of dementia that this visual metaphor reflects?

## Method

### Study procedure

Fifty-one people from the Midlands, UK, were recruited through local support, care, advocacy and activity groups. As Table 1 shows, participants’ ages ranged from 21 to 87 (mean age: 64.3), with 29 individuals identifying as women and 22 as men. Seventeen people had a dementia diagnosis, and one person had a mild cognitive impairment that she regarded as pre-dementia. Thirty-three participants without dementia identified as carers and/or family/friends, of whom three were ex-carers. Although types of dementia and time since diagnosis were not explicitly requested, Alzheimer’s disease, vascular dementia and types of working age dementia were particularly discussed, and conversations indicated that time since diagnosis ranged from <1 to >8 years. Participants reflected typical demographics for individuals who participate in research, namely being adults who were engaged with their local community, with an overrepresentation of white British adults (49 of 51 participants) (Fletcher, 2019). Sessions ran from October 2019 and ended abruptly in March 2020 due to national lockdown, so strategies to reach broader demographics were never realised.

**Table 1.** Summary of participant demographics by group.

*Group/Interview*	Age range	Identified gender Men	Women	Person with dementia^a^	Carer	Family/friend	Previously a carer	Total number of people
Group 1	21–83	5	3	3	4		1	8
Group 2	54–61		4			4		4
Group 3	69–80	2	2	2	2			4
Group 4	52–78	2	2	1	3			4
Group 5	26–31	2	1			2	1	3
Group 6	63–87	1	4	2	2	1		5
Group 7	73–87	3	2	3	1	1		5
Group 8	48–64	4	6	4	4	2		10
Interview 1	62		1			1		1
Interview 2	73		1		1			1
Interview 3	84		1	1			1^b^	1
Interview 4	27, 61	2		1		1		2
Interview 5	72		1		1			1
Interview 6	71		1	1				1
Interview 7	69	1					1	1
**Total**	**21–87**	**22**	**29**	**18**	**18**	**12**	**4**	**51**

^a^This includes one participant in Group 7 with a mild cognitive impairment, which she regarded as pre-dementia.

^b^Following caring for her husband with vascular dementia, this individual now has Alzheimer’s disease herself, and is therefore included in both categories.

All participants received detailed information about the project before choosing to do an in-person focus group or individual/pair interview. Location was agreed according to individuals’ needs. Eight focus groups and seven interviews were conducted, ranging from 45 to 105 min in length. These were semi-structured and could be broadly divided into three main stages: (1) discussion about participants’ experiences of dementia, charities and the media, including how participants would explain dementia to others; (2) responding to twenty images used to represent dementia; and (3) responding to different language choices in relation to dementia. Stages (1) and (2) were always conducted, while (3) was occasionally excluded according to the needs of participants.

### Ethics and consent

Ethical approval was obtained from the University of Nottingham’s Faculty of Arts full committee review. Consent was regarded as an on-going process rather than a one-off procedure (Dewing, 2008; Samsi & Manthorpe, 2020). On the day, the study was discussed before obtaining written formal consent. No formal assessments of capacity were carried out, with informal conversation and non-verbal cues being used to determine that participants understood the nature of the research and were keen to take part. On this basis, people were deemed to have capacity to decide for themselves whether they wished to take part. Participants with dementia gave written and verbal consent when they clearly had the capacity to do so; otherwise, in the case of the latter, carers were present and could also be consulted on whether they thought the person was willing to participate. The consent process was tailored to suit individual needs, in an attempt to include, rather than exclude, people with dementia with different communicative needs (for more on the exclusionary consequences of current ethical procedures, see Fletcher, 2020).

As the facilitator, I was mindful of ‘possible expressions of verbal, non-verbal and behavioural displeasure, disengagement and discomfort’ and responded to participants’ needs throughout (Samsi & Manthorpe, 2020, p. 4), including through encouraging refreshment breaks and rescheduling where needed. Participants took part on a voluntary rather than paid basis. They were offered refreshments or meals according to time of day, as well as being given five British pounds as a token of gratitude and a thank you note with the researcher’s contact details following the session. People could opt-in to hear about the study’s findings and were encouraged to contact the researcher with any questions or concerns.

All participants are anonymised and given sequential two-character pseudonyms in this paper. For transparency reasons, letters refer to participants with a form of dementia (e.g. PA), and numbers refer to participants without dementia (P1).

### Stimuli

Stimuli were selected from existing literature on images (Brookes et al., 2018; Harvey & Brookes, 2019), language guidelines for writing about dementia, and a separate multimodal corpus of British charity and news communications between 2017 and 2019 collated by the author. Search engine results and materials that the researcher engaged with spontaneously (namely a promotional charity envelope) were additionally used to complement existing stimuli, with the aim of engaging with a broad range of dementia representations (e.g. Alzheimer Europe, 2013; Van Gorp & Vercruysse, 2012).

The visual metaphor analysed here is a stock image (Shutterstock, 2019) that, alongside variations of the image, is widely distributed. The autumnal image was presented to participants on the third of five pages of images, each arranged in sets of four. The autumnal image and its three accompanying ones can be termed ‘hybrid metaphors’, since multiple phenomena are visually represented as interacting in the same space in a way that would be impossible physically, which subsequently establishes new meanings beyond each individual component (Forceville, 2008, p. 465). In the other images on this page, a brain blurs and disintegrates into pixels, the back of a man’s head fragments, and a woman loses a jigsaw piece from her head, leaving a dark void where it should be. All four images share a common discourse of loss and degeneration, which is set against the following page of literal images orienting around the living well discourse (Morgan, 2018), showing people looking happy in social situations, getting married and Terry Pratchett holding a sign to express that it’s possible to live well with dementia and still write a bestseller.

The autumnal metaphor emerged as a particular point of interest in the participants’ discussions, since it was one of the most positively evaluated overall yet was simultaneously resisted by several participants who associated it with inaccurate and harmful depictions of people with dementia. It is therefore a particular site of tension between different conceptions of dementia. The image realises multiple metaphors, most obviously THE BRAIN IS A (DECIDUOUS) TREE and DEMENTIA IS LOSS/DECAY, as well as the synecdoche whereby the head/brain represents the whole person. Much of this is made explicit in the stock image keywords (Shutterstock, 2019, my emphasis):

*Brain aging* and *memory loss* due to Dementia and Alzheimer’s disease with the medical icon of a group of color changing autumn fall trees in the shape of a *human head losing leaves as a loss of thoughts and intelligence function*.

Participants were not privy to these keywords, being given only the image and the context of dementia representations. Overall, this visual metaphor holds great potential for analysis: it is popular, has complex meaning potential and is controversially received by participants.

### Analytical approach

This paper takes an inductive, qualitative approach to the data, drawing in particular on thematic discourse analysis, which, as with any flexible approach with no set formula, requires a definition of how it is used in this context (Cheek, 2004). Here, I focus on how participants linguistically orient themselves in relation to the aforementioned visual metaphor and its associated discourses, collating these responses into three main themes. Manual transcription enabled familiarity with the data, after which themes were generated through repeated close reading and cross-comparisons. In a departure from more traditional thematic discourse analysis approaches (Moran & Lee, 2018; Peel & Harding, 2014; Singer & Hunter, 1999), I additionally incorporate my own analysis of the image to help contextualise, and expand, participants’ responses. This is greatly influenced by a social semiotic theory of communication, and of multimodal critical discourse analysis in particular, which examines and challenges the ways in which different semiotic resources, here a visual metaphor, reproduce and naturalise specific views of the world (see Brookes et al., 2021).

Since my positioning influences the direction of these findings, both through my relationship with participants and approach to analysis (Finlay, 2002), it is important to clearly outline my stance. I regard dementia as a complex bio-psycho-social condition (Sabat, 2014), and as a disability more than a disease, in which a range of relational, social, political, environmental and economic variables can (dis)empower people who have dementia (Shakespeare et al., 2019). This stance underpins both the project and this paper.

Here, I structure analysis thematically rather than cross-comparing different participant groups. Ideologically, this mitigates unnecessary distinctions between people with and without dementia, while practically, it enables richer, more nuanced analysis within each theme. Instead, the two-character pseudonym system outlined above clearly identifies individual participants and whether they have lived experience of dementia to provide greater detail and transparency for readers.

### Analysis

The subjectivity of metaphor interpretation is evident in the range of responses to the same visual metaphor. This is epitomised by one focus group’s debate, where PM asserts that the image is ‘true’, while PK equally confidently responds that ‘no it’s not true’. Elsewhere, PC stresses that ‘it’s got to be a personal thing everybody’s dementia’s different like everybody’s fingerprint is different’. These varying and subjective responses are organised below into three key themes: (1) interpretations that the metaphor does not depict dementia; (2) that it is meaningful for accurately explaining brain deterioration; and thus (3) that it reinforces inaccurate and/or ‘hopeless’ discourses of what having dementia involves and thus requires changing.

### Not dementia: ‘It’s a bloody tree’

Despite being told that this and the other stimuli are used to in relation to dementia, some participants situate the image as outside this context. Two people achieve this through resisting any metaphorical reading of the image, which cautions against assuming everyone to engage at the connotative level for metaphorical images. PH rejects discussing any target domain, stating that ‘being a physicist, it’s a bloody tree’, an identity marker that he consistently associates with wanting to focus on what a picture shows, rather than suggests. Meanwhile, PA rather poetically situates it as ‘just a pretty picture, and if you don’t know what it’s about, and you can’t read it, in the field or whatever, you just drive past.’ These different responses demonstrate the role of context and personal stance in interpreting visual metaphors.

Other participants highlight that the visual metaphor is not constrained to a dementia context; P33 describes it as ‘not so clear’ as other visual representations, joking that it could be about hair loss, while PN initially declares that ‘it’s how. most. people get eroded I mean, it could be a child couldn’t it. if the child is starved of imagination and. love and care.’ Despite situating the image in non-dementia contexts, both accounts clearly engage with the image as a means of metaphorically conveying loss, with P33 positioning it as external (loss of hair) and PN as internal (a wearing down of a person through their environment). PN’s reading is particularly striking, as she draws on another source domain, erosion, to position people of all ages as vulnerable to the effects of their surroundings, in which adverse exposure (here to a lack of care, love and imagination) can gradually wear away at individuals. As will become apparent, when in a dementia context, rather than considering the role of a person’s social environment, the eroding force is attributed to *internal* changes from the condition, both in these interviews and in published written works (see Zimmermann, 2017, p. 80).

### Meaningful presentation of dementia: ‘It explains what happens’

The majority of participants praise the visual metaphor for being ‘meaningful’ (P11, P28) in its explanation of ‘how dementia affects your brain’ (P21). It is consistently described in terms of showing (‘you can *see* what’s happening’: PG) and explaining (‘I’m going through it myself and. Every time you – *looking* at this, it *explains* what happens to your brain’: PJ) (my emphasis). For some participants, this image is described as the best of all the twenty stimuli shown to them ― P22 states that ‘that to me explains more to me than anything else in the book’. What is it about this visual metaphor that makes it so compelling?

Immediately obvious is the image’s varied and saturated (brighter) colours, which are widely associated with exuberance (Ledin & Machin, 2018a). Its colours are described as ‘nice’, and as ‘happier’ and ‘more gentle’ than ‘the dim’ images that it sits alongside (P22), which use predominantly beige and grey hues, elsewhere critiqued for their ‘quite cold […] stark background’ (P19). However, most responses orient not around colour palette but around the visual metaphor being a useful resource to help explain internal, complex biological changes that are usually invisible to the human eye. Like brain scans, this visual metaphor is positioned as showing what human eyes cannot see without technology: the brain and its processes. P2 even positions the metaphor as ‘*ideal* for saying what a brain does what’s happening when you’ve got dementia’ (my emphasis). Unlike brain scans however, this metaphor is not regarded as an ‘expert image’ that remains inaccessible to non-experts (Dumit, 1999). Instead, the image is praised for being ‘done simply’ (P23), reinforcing the explanatory power of metaphorically mapping more tangible source domains onto dementia.

The visualisation of internal biological processes is evident even when the image is discussed solely in terms of this metaphor’s target domain (the head/brain) without mentioning the source (the autumnal tree). In an interview with PF, who has Alzheimer’s disease and cared for her late husband when he had vascular dementia, I am told that:

The one with three heads, um, I take it or I feel, that that’s [the green head], very imperceptibly starting, and then the goldie one is, the tangles are getting worse and the nerve endings are not going through and giving you the right coordination, and then the red one is when it’s got destroyed a lot, the latter stages.

Here, PF refers to ‘heads’ as the salient feature, not trees. Similarly, other participants say ‘faces’ (PI, P20, P22), reflecting the extent to which this hybrid metaphor merges trees with human heads (Forceville, 2008). PF positions the heads as a means of seeing internal biological changes, even when they are ‘imperceptibl[e]’ externally. Drawing on medical terminology to refer to sites of change (‘tangles’ and ‘nerve endings’), PF foregrounds affected ‘coordination’ as an obvious external manifestation (notably different to the usual focus on changes to memory). Significantly, PF’s account metaphorically presents dementia as a progressive destruction of the brain (which falls within DEMENTIA IS LOSS), whereby the red face has ‘got destroyed a lot’ in ‘the latter stages’ of life with dementia. This reflects the image’s increasing loss of leaves, which is concentrated in the brain area of these tree-heads ― by the third, red tree, the brain has very few leaves remaining.

Many individuals explicitly connect the trees’ seasonal changes to the brain with dementia through loss. Notably, PM, who has a mild cognitive impairment regarded as pre-dementia, likens a tree losing an increasing number of leaves in autumn/winter to a brain losing cells during dementia:

That’s why these pictures are good because you’re suddenly normal, and then you start, to lose more, like a tree, when it’s, it’s losing its leaves in the wintertime […] and then you lose some, like in the wintertime your tree will lose some leaves, and then it will lose more, and then it will lose more, and that’s just like your brain. It loses more, and more, brain cells.

That PM repeats ‘lose/losing’ seven times reflects the extent to which loss is foregrounded. The concept of loss is referred to throughout accounts, both directly (P22, P23), and via a range of partially synonymous terms, including: ‘it’s deteriorating’ (P22), ‘everything’s fading away, slowing down, degrading’ (P28), ‘it’s […] dying off’ (P28, P30) and ‘the brain disappears’ (P2). The terms ‘fading away’ and ‘disappears’ are particularly reminiscent of other metaphors that more explicitly associate cognitive losses during dementia with a loss of self, particularly dementia being a living death, through which people are positioned as fading/faded and sub-human (Aquilina & Hughes, 2006; Behuniak, 2011; Van Gorp & Vercruysse, 2012).

Some participants suggest that the autumnal visual metaphor softens the underlying discourse of loss and degeneration, as it is ‘showing you without the kind of human expression’ present in other representations (P17). For P10, that ‘it’s not an actual human face’ makes it feel ‘less […] like the person’s humanity is disappearing’. Through ‘less’, P10 acknowledges that the tree-head metaphor mitigates, rather than avoids, this implication. This inference is reflected in participants’ responses; in another focus group, P23 explains that ‘you’ve got a whole tree, and then it’s lost a bit, and then it’s lost a bit more’. Here, the emphasis is on ‘a whole tree’ that increasingly loses ‘a bit more’, thus becoming incomplete. Indeed, the subsequent tree-heads are very literally *less than* the first one, since their leaves/brain cells lie on the floor below them. When the tree-head is mapped onto a person with dementia, the implication becomes that an increasing loss of brain cells shifts the person ever further from being a ‘whole’.

Many participants also appreciate the brain as having ‘deteriorated *over time*’ (P22, my emphasis). People consistently emphasise the slowness of the progression, often referring to ‘gradual/gradually’ (PN, P2, P7) and ‘slowly’ (PI, P28). P14 interprets it as meaning a ‘steady decline […] Because Alzheimer’s is a steady decline’. Alternatively, P2 contextualises the seasons metaphor not as stages of dementia but in terms of the life cycle, suggesting that the image shows that ‘you start off young and vibrant and then gradually your brain starts to go and then at the end your brain’s gone’. This speaks to a core aspect of the seasons metaphor when it ends with winter ― there is no spring, no renewal. The image achieves this sense of time through placing three different stages alongside each other, since ‘you trace it from the beginning’ (P28) and ‘you can see how it’s going along’ (P21). Moving across the page in this way reflects a Western metaphorical mapping of movement onto time; here time is stationary, frozen into three moments, and (assuming Western cultures’ left to right reading direction) we as viewers move through it, towards the future, as signified by the red head (Lakoff & Johnson, 1980, p. 44).

This movement from the green head on the left to the red on the right accrues additional significance when considered in relation to the trend noted by Kress and van Leeuwen (2006), that for those who read left to right, information on the left is positioned as the already familiar, or ‘given’ (i.e. normal), while information on the right is less familiar, or ‘new’ (180). According to such a theory, the green head on the left is positioned as ‘the normal person’ (PN) and moving right reflects not only time passing but a shift away from the familiar to the unfamiliar, abnormal state of increasingly progressive dementia. Returning to the example of PM, the green tree with full foliage is explicitly established as representing when ‘you’re normal’. That it is ‘you’, rather than ‘your brain’ that is normal here reflects a wider cultural conflation of the brain with the whole person, which will be explored further in the third analytical theme.

### Inaccurate representation: ‘Dementia doesn’t quite work like that’

In the three focus groups where members resisted the visual metaphor, participants present the multiplicity, individuality and changeability of dementia as their key counter discourses, challenging and seeking to adapt the DEMENTIA IS LOSS metaphor and the brain as person synecdoche, while seeming to accept THE BRAIN IS A TREE. The contention surrounding the visual metaphor is epitomised by the following exchange between two members of a dementia-specific Public and Patient Involvement (PPI) group:

PK Dementia doesn’t quite work like that does it, it’s not a straight line. […]

PM That’s why these pictures are good because you’re suddenly normal, and then you start, to lose more, like a tree, when it’s, it’s losing its leaves in the winter time

PK But they point, as three stages

PM Yeah but it is stages

PK You could have a forest, of those trees

Having examined PM’s contribution earlier, the focus here is on PK’s two main criticisms of the image’s underlying discourses. First, recognising the linearity of the direction of time (left to right) in the image, PK emphasises that dementia is *not* a linear decline, expressed metaphorically as ‘not a straight line’. Second, he resists the ‘three stages’ as an oversimplification of the reality of dementia, suggesting instead that a ‘forest’ of trees would better represent its manifold nature.

Participants consistently critique the linearity and finality of the image’s depiction of progression with dementia. P1, whose late wife had dementia, argues that it ‘apparently shows a constant deterioration whereas if you’ve been with someone who has dementia you know that that’s not the case’, as there are ‘moments of lucidity and those moments it’s as if everything is restored, even the memory, and you can’t cope because within a second it’s […] gone.’ Rather than a forest, P1 suggests altering the image to show ‘leaves floating around because it can always come back’. The group supports this as a more accurate portrayal and later, P3 returns to P1’s idea of incorporating changeability, adapting the tree metaphor accordingly:

it’s just like what [P1] said about how the leaves, they do gravitate around the tree and sometimes they come back, you know and I think that’s very true and I think [this image] kind of take[s] away from that silver lining […] the leaves do stay around the trees you know sometimes they settle back down, sometimes they blow away

P3 touches on a flaw in the autumn to winter metaphor for trees here ― that leaves, once fallen, cannot reattach. He therefore extends the conceptualisation of what it means to be lost, highlighting that leaves ‘do gravitate around the tree’ and can ‘come back’ or ‘settle’, separating this from the permanent loss of ‘blow[ing] away’. This ability for things to return is the ‘silver lining’ that he suggests the image’s linear degeneration is currently missing. Similarly, P5 rejects the ‘hopeless’ image as not matching her individual reality, since ‘dementia in my case, I can only speak personally, it’s not hopeless to me. It’s difficult sometimes, it’s funny sometimes and that’s just portraying the brain decaying’. She rejects the decay metaphor in favour of a machine one, arguing that ‘your brain’s not decaying, it’s just not working properly. in different areas’.

In another focus group, P18, whose grandmother had dementia, instead recommends varying the areas experiencing leaf-loss (i.e. ‘not the same part […] missing’) to reflect flux with dementia. He subsequently shifts the focus to identity, arguing that:

You never know what you’re going to get. Like when I go visit my grandma’s like I’m either going to get the grandmother, I grew up with, I’m going to get the grandmother, my mother grew up with so it’s like. But that’s still them. You just get a different part of them when you visit.

Extending the changeability of life with dementia, where a ‘missing’ ‘part’ can return to people, P18’s account positions the person themselves as a constant (‘that’s still them’), and just as presenting ‘a different part of them’ depending on the day. Similarly, P3 calls for a recognition of the personal aspect of our abilities and identities to challenge the metaphor’s underlying discourse of loss:

the person that we all know or whatever isn’t leaving, they’re not disappearing […] It’s not as simple as that, it’s very hit and miss, come and go, very personal, very built within that relationship or that person not just that it’s gone.

Here, P3 emphasises the personal and relational aspects of identity, emphasising that identities are ‘built within’ our relationships and beings, and therefore cannot be ‘disappearing’. Both P3 and P18 strongly resist the underlying discourse of loss of self, instead presenting an alternative, more nuanced conceptualisation whereby people have plural and fluctuating identities that are in part held by their relationships with the people around them (Hughes, 2014). It is in these counter discourses that participants highlight that people with dementia are far more than their progressive biomedical condition, challenging discourses of loss (Bryden, 2020), and offering creative solutions to better match the visual metaphor to their experiences of dementia and identity.

## Discussion

The foregoing analysis demonstrates that metaphor interpretation in relation to dementia is subjective and context dependent (Castaño, 2020). This particular visual metaphor contributes a novel case study into the range of responses that can be expressed amongst even a small, fairly homogenous group of people (here, mainly white working/middle class British people actively engaged with their community in the Midlands). Participant responses indicate that this autumnal metaphor can be helpful for understanding and communicating an inherently complex and abstract condition ― notably, it is frequently positioned as *showing* and *explaining* what happens to people’s brains in an accessible way. Yet, the same visual metaphor can also be unhelpful, in the first theme because people find the metaphorical reading within the image inaccessible, unnecessary or vague. In the third theme, it is resisted for inaccurately depicting dementia as it incorporates neither nuances such as flux within the progression of the condition nor the potential for experiences other than hopelessness. For other participants, it fails to acknowledge a more holistic understanding of what personal identity means, both for individuals with dementia and for people more broadly, constraining the focus to ‘brainhood’ (Vidal, 2009) rather than considering the relational aspects of our multifarious and ever shifting identities (Hughes, 2014; Sabat, 2018). The range of responses speak to a tension at the heart of metaphor, namely that mapping a simpler domain onto a complex one can make abstract things more concrete and understandable, but at the risk of grossly oversimplifying or misconstruing the target phenomenon in the process.

This visual metaphor provides a useful site for studying competing discourses, since participants’ evaluations are entwined with their positioning in relation to prominent dementia discourses, namely of a biomedical conceptualisation of dementia as loss and the prioritisation of the brain as the locus of identity. A critical analysis of this metaphor exemplifies that a socially acceptable representation that is not explicitly disempowering can nonetheless express damaging underlying ideologies that position people with dementia as lesser and abnormal. Researchers argue that a biomedical model of dementia normalises the DEMENTIA IS LOSS metaphor through its positioning of people as patients ‘whose brain has been destroyed by the disease and who therefore no longer exists as a person but only as a body to be managed’ (Behuniak, 2011, p. 74). We see this in participants’ focus on the brains of people with dementia being ‘destroyed’ (PF) or disappearing. Yet these participants are clearly responding to the specific biomedical context of this image, since, although not the focus of this paper, elsewhere the same people draw on person-centred, embodied, social and/or relational understandings of dementia (see Hughes, 2014; Kitwood, 1997; Kontos, 2006; Sabat, 2018). This observation highlights the extent to which different, sometimes conflicting discourses can compete at a micro conversational level, as well as at the macro level of society.

Context and multiplicity therefore become essential considerations, both for this paper and for visual representations more generally. The diversity of the above responses reinforces that ‘images only become meaningful in the context of their viewing, and as such do not “carry” precise or universal meanings that can be read from them’ (Pink, 2011, p. 268), which has a range of methodological, theoretical and practical implications. Firstly, this study hopes to have contributed convincing evidence for the value of combining critical researcher analysis with the responses of a range of individuals who have different experiences and worldviews, particularly when the researcher does not have lived experience of the condition being represented. Relatedly, the contextual nature of interpretation raises the question of how different research contexts (including different researchers and participants) would influence findings, since these responses are specific to particular settings and researcher/participant dynamics. Notably, outside of the project context, other individuals who engage with the image highlight to me that there is an inevitable return to spring in the seasonal cycle, arguing either that this misconstrues the finality of dementia or offers a more hopeful, non-linear narrative. The role of context cautions against any ‘universal’ metaphor readings being applied to the larger community of people affected by dementia. Equally, this analysis does not consider how someone’s specific experience with dementia (e.g. type of dementia, lived or indirect) might influence the reception of this visual metaphor or representations more broadly. These are all points for future research, to enable further nuance.

This visual metaphor is analysed in a social context in which metaphors for dementia are often disproportionally loss-oriented, creating a disempowering and stigmatising environment for people with dementia (Alzheimer Europe, 2013; Van Gorp & Vercruysse, 2012). Therefore, this paper adds to calls for greater nuance and balance in metaphorical representations, to provide people with a means of expressing suffering and loss *alongside* other aspects, including more positive ones (Bartlett et al., 2017 ; Castaño, 2020). Notably, participants’ critiques support the need for more holistic models of selfhood, as the hegemony of biomedical and brain-oriented models fails not only people with dementia but humanity more broadly.

Since metaphors shape collective understandings of dementia, they are a powerful resource to help develop and extend such understandings (Zeilig, 2014a). This can involve platforming lesser-known metaphors to develop our cultural script, as with Oliver’s (2016) blog post that ruminates on four personal metaphors for his experience of dementia: a ‘fog’, ‘hole punch’, ‘being a swan’ and ‘mining’. This social shift can also involve reclaiming and adapting existing well-known metaphors. Examples of this are growing; Castaño (2020) observes bloggers with dementia discussing their experiences in terms of a *transformation* rather than loss of self. Likewise, the ‘empty shell’ metaphor can be revised to position people with dementia as not empty but containing inner pearls, emphasising that effort and skill is needed to reach and connect with each individual, much like an oyster’s hidden pearl (Anderson, 2012, in Alzheimer Europe, 2013). In this study, participants creatively develop the visual metaphor shown to them, including by suggesting a forest of trees, allowing leaves to reattach or remain in proximity, and changing the areas missing leaves so that the linear cognitive decline currently pictured can instead become a flux of different abilities and identities. In this spirit of modification, I invite readers to consider alternative ways of approaching the autumnal tree metaphor. Returning to PF’s metaphor of erosion, what is the role of the environment in the loss depicted? Is there agency to be gained in still standing (Zimmermann, 2017, p. 80)? Could the opportunity for re-growth in spring signal transforming identities?

## Conclusion

This paper demonstrates the subjectivity of people’s responses to a single visual metaphor, on multiple discursive levels. While metaphorically mapping trees onto the brain appeared largely helpful, envisioning people as brains, and dementia as loss, were more problematic. The aim here has been to stimulate discussion of the role of visual metaphors in expressing and comprehending dementia, and to showcase the importance of attending to nuance and subtler manifestations of stigmatising discourses moving forward.
